# Impact of resident versus specialist performed cervical spine surgery

**DOI:** 10.1016/j.bas.2025.105864

**Published:** 2025-11-03

**Authors:** Tobias Overmark, David Kocemba, Tim Damgaard Nielsen, Joel Borgstedt-Bendixen, Mikkel Mylius Rasmussen

**Affiliations:** a Department of Neurosurgery, Aarhus University Hospital, Aarhus, Denmark; b Department of Clinical Medicine, Aarhus University Hospital, Aarhus, Denmark; c Private Hospital Mølholm, Vejle, Denmark

**Keywords:** Cervical spine surgery, Anterior cervical discectomy and fusion, Resident, Patient reported outcome, Postoperative pain, Surgical education

## Abstract

**Introduction:**

Cervical spine surgeries are complex procedures associated with a significant risk of suboptimal clinical outcomes. In Denmark, residents perform cervical spine surgery under specialist supervision during their training. This study aims to assess whether patient-reported outcomes (PROs) differ between cervical spine surgeries performed by supervised residents and those performed by specialist neurosurgeons.

**Research question:**

To compare PROs following cervical spine surgeries performed by residents versus specialist neurosurgeons.

**Materials and methods:**

Data from 464 surgical decompressive procedures performed at Aarhus University Hospital between 2018 and 2023 were extracted from the national spine surgery database, DaneSpine. Univariate and multivariate ordinal and binary logistic regression were performed to calculate odds ratios (OR) for lower arm and neck pain (visual analogue scale), lower neck disability index (NDI), and patient satisfaction at 1-year follow-up. Specialist neurosurgeons served as the reference group.

**Results:**

Multivariate ordinal and logistic regressions found no statistically significant difference in PROs between the groups. At 1-year follow-up, we find a tendency towards patients with resident-performed surgery having a better chance of improvements for arm pain (OR 1.05, CI = 0.69–1.58), however a lower chance of improvement for neck pain (OR 0.93, CI = 0.63–1.39), NDI (OR = 0.80, CI = 0.49–1.31) and being satisfied (OR = 0.89, CI = 0.55–1.46).

**Discussion and conclusion:**

Our results support the practice of graduated surgical responsibility: with proper supervision and case selection, resident surgeons can safely participate in cervical spine surgeries without significantly affecting patient-reported outcomes.

## Highlights

• Resident-performed spine surgery shows outcomes similar to specialists.
• No significant difference in pain or function at 1-year follow-up.
• Patient satisfaction remained high regardless of surgeon level.
• Resident involvement did not worsen disability after cervical surgery.
• Supports safe inclusion of residents in complex spine procedures.

## 1 Introduction

In teaching hospitals, balancing surgical education with optimal patient care is an ongoing challenge. As residents progressively assume greater surgical responsibilities, their involvement may influence patient outcomes. This balance becomes particularly important in technically demanding procedures, such as anterior cervical discectomy and fusion (ACDF) or cervical decompression through a posterior midline approach, where technical precision is required to restore function and relieve pain (Kirzner et al., 2018; Hsueh et al., 2023; Nassr et al., 2009; Butler et al., 2019).

Cervical spine surgeries are complex procedures, with patient-reported outcomes (PROs) being influenced by surgical decisions and perioperative care (Yang et al., 2024; Debono et al., 2019). While prior studies have generally found resident involvement to be safe in various spinal procedures (Phan et al., 2019; Kim et al., 2016; Patel et al., 2022; Lau et al., 2020), only few have evaluated how supervised resident-performed cervical surgeries compare to those performed by specialist neurosurgeons with respect to long-term PROs, such as pain relief, functional recovery, and satisfaction (Patel et al., 2022; Ells et al., 2025). Existing literature has focused on perioperative complications or short-term outcomes (Kim et al., 2016; Lee et al., 2018; Seicean et al., 2018), leaving a gap of knowledge regarding the clinical impact of resident-performed surgeries.

In Denmark, supervised residents routinely perform cervical decompressions, yet there is limited empirical data on whether this practice affects patient outcomes. Addressing this knowledge gap is important, for the dual purpose of ensuring patient safety and transparency as well as quality testing the current teaching protocols.

In this study, we aim to evaluate whether supervised resident-performed cervical decompression surgeries for degenerative cervical nerve root or spinal canal stenosis result in different one-year PROs compared to those performed by specialist neurosurgeons.

## 2 Methods

### 2.1 Study population

Patients who underwent cervical spine surgery at the Department of Neurosurgery, Aarhus University Hospital, from 2018 to 2023 with procedure codes KABC30 (decompression of cervical nerve-root), KABC50/KABC60 (decompression of cervical spine and nerve-root) and KABC21/KNAG40 (anterior cervical discectomy and fusion) were included.

### 2.2 Patient data

We utilized information from DaneSpine, which consists of the surgeon's technical reports, and prospectively collected PROs based on a questionnaire at baseline and 1-year follow-up. The surgeon's report includes American Society of Anesthesiologists (ASA) classification (Hendrix and Garmon, 2025), surgery type, levels of the surgical field and perioperative complications. PROs include socio-demographic data, the Visual Analogue Scale (0–100) (Briggs et al., 1999) for arm pain (VAS-AP) and neck pain (VAS-NP), the Neck Disability Index (NDI) (Vernon and Mior, 1991), duration of arm and neck pain, the European Myelopathy Scale (EMS) (Herdmann et al., 1994) and satisfaction status. Satisfaction status was assessed at 1-year follow-up. Patients were asked whether they were satisfied with the outcome of their surgery. Response options included: satisfied, undecided, and dissatisfied.

### 2.3 Exposure parameters

The surgeon's level of experience was categorized as either a resident or a specialist neurosurgeon. The neurosurgical residency in Denmark consists of 6 years of training. Cervical spine surgeries are rarely performed by a first-year resident as the primary surgeon. After the first year, ACDF surgeries will routinely be performed by trained residents, listed as primary surgeons, and supervised by a specialist neurosurgeon. The training mostly follows the “see one, do one, teach one” principle. Mandatory courses and training on cadavers accompany this.

Surgeon identification was retrieved from patient files. A surgeon may be registered as either supervisor, primary surgeon or assistant. Priority was given to whoever was recorded as the primary surgeon. The listing as primary surgeon in the patient files is assigned to the surgeon responsible for the majority of the surgery, including approach, decompression, and instrumentation. If multiple surgeons were listed as primary, precedence was assigned to the surgeon who performed the decompression. This information was found in the medical records. In cases, where a complication necessitated intervention by a colleague, the initial surgeon responsible for the complication was prioritized. If multiple primary surgeons were listed in the patient files with missing information on who performed the decompression, the specialist neurosurgeon was considered the primary surgeon.

### 2.4 Statistical analysis

The primary outcomes were VAS-AP and NDI at 1-year follow-up. The secondary outcomes were VAS-NP and patient satisfaction at 1-year follow-up. Patients with missing PROs data were excluded from analysis (Supplementary A). To address missing follow-up PRO data, we made a sensitivity analysis using multiple imputation with predictive mean matching. We assumed data was missing at random. This assumption was supported by variables predictive of missingness included in the imputation model. No strong evidence suggested that patients with poorer outcomes were systematically less likely to respond at follow-up (Andersen et al., 2024) (Supplementary B). Possible confounders were ordered by presumed clinical relevance before analysis and included based on this order (Supplementary C).

Exploratory analysis of continuous PROs indicated multimodal distributions (Supplementary D). We followed recent recommendations (Heller et al., 2016) and applied a continuous ordinal regression model to preserve the continuous and ordinal properties of the VAS scale (Manuguerra et al., 2010, 2020). For NDI, a logistic ordinal regression model was used after categorizing NDI scores into clinically meaningful groups: none (0–4), mild (5–14), moderate (15–24) and severe to complete (25–50) (Vernon, 2000; MacDermid et al., 2009). For satisfaction, which was recorded as a binary outcome: satisfied, undecided/dissatisfied, we used a binary logistic regression model. For all models, surgery performed by residents was compared to surgery by specialist neurosurgeons, and the latter served as reference.

Model diagnostics showed no evidence of multicollinearity or significant interactions between clinically relevant covariates. Linearity assumptions for continuous variables were satisfied, and the proportional odds assumption was tested and confirmed for ordinal predictors, including educational level. (Supplementary E-H). A minimum of 10 events per variable was required in adjusted logistic models. In continuous ordinal models, we had 18 observations per degree of freedom.

For baseline comparisons (Table 1), chi-squared tests were used for categorical variables, and Fisher's exact test when expected cell counts were below 5. A Student's t-test was used for continuous variables with normally distributed residuals and equal variances; otherwise, the Wilcoxon rank-sum test was used.

**Table 1:** Baseline and surgical characteristics.

Characteristics	Total (*n* = 464)	Surgery performed by specialist neurosurgeons (*n* = 370)	Surgery performed by residents (*n* = 94)	P value
Gender				0.02^+^
Male	253 (55 %)	191 (52 %)	62 (66 %)	
Female	211 (45 %)	179 (48 %)	32 (34 %)	
Age	56 (48–64)	56 (48–64)	54 (46–64)	0.51^‡^
BMI	27.1 (24.1–31.0)	27.2 (24.3–31.2)	26.5 (23.6–30.9)	0.10^‡^
Smoker	106 (23 %)	81 (22 %)	25 (27 %)	0.41^+^
Diabetes	33 (7.1 %)	26 (7.0 %)	7 (7.4 %)	>0.99^+^
VAS-AP (0–100)	60 (36–79)	63 (40–79)	51 (22–78)	0.04∗
VAS-NP (0–100)	62 (31–80)	64 (37–81)	49 (20–78)	0.04∗
NDI (0–50)	20 (14–27)	20 (14–26)	21 (12–27)	0.83∗
Arm pain duration				0.64^+^
<3 months	69 (15 %)	51 (14 %)	18 (19 %)	
3–12 months	195 (42 %)	156 (43 %)	39 (41 %)	
12–24 months	98 (21 %)	80 (22 %)	18 (19 %)	
>24 months	98 (21 %)	79 (22 %)	19 (20 %)	
Neck pain duration				0.57^+^
<3 months	91 (20 %)	71 (19 %)	20 (22 %)	
3–12 months	143 (31 %)	110 (30 %)	33 (35 %)	
12–24 months	87 (19 %)	70 (19 %)	17 (18 %)	
>24 months	138 (30 %)	115 (31 %)	23 (25 %)	
European Myelopathy Scale grade				0.22^†^
No myelopathy	133 (31 %)	112 (33 %)	21 (23 %)	
Grade I	252 (58 %)	194 (56 %)	58 (64 %)	
Grade II	50 (11 %)	38 (11 %)	12 (13 %)	
Grade III	0 (0 %)	0 (0 %)	0 (0 %)	
Decompression surgery types				0.12^†^
KABC30	45 (9.7 %)	41 (11 %)	4 (4.3 %)	
KABC50/KABC60	58 (13 %)	47 (13 %)	11 (12 %)	
KABC21/KNAG40	361 (78 %)	282 (76 %)	79 (84 %)	
Number of levels				0.46^†^
1 level	340 (73 %)	270 (73 %)	70 (74 %)	
2 levels	110 (24 %)	87 (24 %)	23 (24 %)	
3 levels	14 (3.0 %)	13 (3.5 %)	1 (1.1 %)	
ASA				0.70^†^
1	138 (30 %)	110 (30 %)	28 (33 %)	
2	259 (56 %)	209 (56 %)	50 (53 %)	
3	65 (14 %)	49 (13 %)	16 (17 %)	
4	2 (0.4 %)	2 (0.5 %)	0 (0 %)	
Prior neck surgery	82 (18 %)	72 (19 %)	10 (11 %)	0.06^+^
Surgery indication				0.85^+^
Radicular arm pain	321 (90 %)	252 (90 %)	69 (88 %)	
Others	37 (10 %)	28 (10 %)	9 (12 %)	

VAS-AP: Visual Analogue Scale for arm pain (0–100); VAS-NP: Visual Analogue Scale for neck pain (0–100); NDI: Neck Disability Index (0–50); ASA: American Society of Anesthesiologists classification. *n* = (%); Median (Q1 – Q3). ^+^Chi-squared test, ∗Wilcoxon rank-sum test, ^†^Fisher's exact test, ^‡^Student's t-test. Superscript (1) Includes radicular paresthesia, paresis and paralysis.

The statistical analysis was performed using RStudio (version 2025.09.1 + 401) with the following packages: ordinalCont, ordinal, MASS, VGAM, MICE, brant, MatchIt, cobalt and tidyverse.

## 3 Results

### 3.1 Baseline demographics

**Fig. 1:**
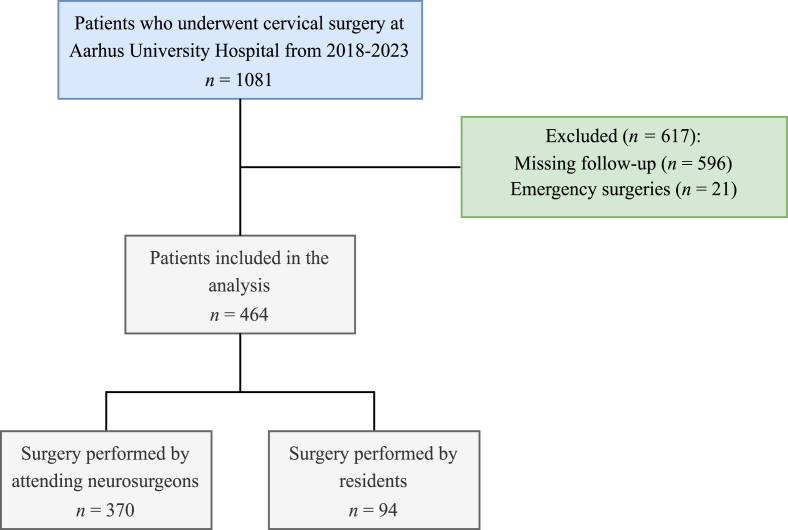
Study population flow chart.

A total of 1081 patients were included. 21 had emergency surgery and 596 did not complete follow-up leaving 464 patients eligible for analysis. Specialist neurosurgeons performed 370 surgeries, while 94 surgeries were performed by a supervised resident (Fig. 1). Median age was 56 (IQR = 48–64), and 55 % were male. Residents performed surgery on more male patients (66 %) compared to specialist neurosurgeons (52 %; P value = 0.02). Patients who underwent surgery by specialist neurosurgeons reported higher median baseline scores for arm pain (VAS-AP: 63 vs. 51) and neck pain (VAS-NP: 64 vs. 49) compared to those treated by residents. Additionally, a greater proportion of patients in the specialist group had a history of prior neck surgery (19 % vs 11 %; P value = 0.06). Three-level procedures were more frequent among specialist neurosurgeons (3.5 %) than residents (1.1 %). Indications for surgery were comparable between the groups. Detailed patient characteristics are provided in Table 1.

### 3.2 Pairwise comparisons of continuous PROs from baseline to 1-year follow-up

Table 2 summarizes pairwise comparisons of continuous PROs from baseline to 1-year follow-up. Significant improvements (P value < 0.01) were observed in VAS-AP, VAS-NP and NDI scores across all groups. Patients operated by residents experienced similar median improvements in VAS-NP (median change: 21 vs. −20), VAS-AP (median change: 24 vs. −26) and NDI (median change: 6 vs. −4) compared to patients operated by specialist neurosurgeons. At 1-year follow-up: 61 (13 % of total) patients reported a VAS-AP of 0, 57 (12 % of total) patients reported a VAS-NP of 0, and 34 (7.4 % of total) reported an NDI of 0 (Supplementary A).

**Table 2:** Pairwise comparison of continuous PROs from baseline to 1-year follow-up.

	Baseline median (IQR)	1-year follow-up median (IQR)	Baseline vs 1-year follow-up	
			Median change	P value
*Overall*				
VAS-AP	60 (35–79)	19 (2–50)	−26	<0.01∗
VAS-NP	60 (30–80)	24 (4–54)	−20	<0.01∗
NDI	20 (14–27)	13 (6–20)	−6	<0.01∗

*Specialist neurosurgeons*				
VAS-AP	63 (39–79)	20 (3–51)	−26	<0.01∗
VAS-NP	62 (36–80)	26 (4–55)	−20	<0.01∗
NDI	20 (14–26)	13 (6–20)	−6	<0.01∗

*Residents*				
VAS-AP	52 (23–78)	13 (2–46)	−24	<0.01∗
VAS-NP	49 (21–78)	16 (3–50)	−21	<0.01∗
NDI	20 (12–27)	13 (6–21)	−4	<0.01∗

PROs: Patient-reported outcomes; IQR: Interquartile range; VAS-AP: Visual Analogue Scale for arm pain (0–100); VAS-NP: Visual Analogue Scale for neck pain (0–100); NDI: Neck Disability Index (0–50). ∗Wilcoxon signed-rank test. Statistical significance is designated by P value < 0.05.

### 3.3 Univariate and multivariate analysis

The univariate comparison of PROs showed no statistically significant differences between groups. Patients operated by residents had 19 % higher odds (OR = 1.19, CI = 0.79–1.77) of achieving a lower VAS-AP, and 17 % higher odds (OR = 1.17, CI = 0.79–1.75) of achieving a lower VAS-NP compared to patients operated by specialist neurosurgeons at 1-year follow-up, from any given baseline VAS-AP and VAS-NP score. For NDI there was a 23 % lower odds (OR = 0.77, CI = 0.50–1.18) for a lower NDI at 1-year follow-up, from any given baseline NDI category and 8 % lower odds (OR = 0.92, CI = 0.57–1.47) for patients being satisfied when compared to patients operated by a specialist neurosurgeon (Table 3) at 1-year follow-up. The continuous ordinal regression models for VAS-AP and VAS-NP were adjusted for ASA, prior neck surgery, myelopathy status, pain duration, number of levels and baseline VAS score. Both models showed no signs of overfitting (Supplementary I). The ordinal logistic regression model for NDI was only adjusted for baseline NDI and myelopathy status to prevent overfitting. The binary logistic regression model for satisfaction was adjusted for the same covariates except neck pain duration and baseline VAS-NP to prevent overfitting. No statistical differences were found between groups in the adjusted analysis, and the OR diminished in all categories except satisfaction. To evaluate influence of the subgroup size difference (94 resident-performed cases vs. 370 by specialist neurosurgeons), we did a propensity score-matched analysis (Supplementary J). The results of this analysis were consistant with our main findings.

**Table 3:** PROs at 1-year follow-up and surgery performed by residents.

	Unadjusted univariate analysis		Adjusted multivariate analysis		
	OR (95 % CI)	P value	OR (95 % CI)	P value	*n*
*Specialist neurosurgeons*	*Ref.*		*Ref.*		
VAS-AP	1.19 (0.79–1.77)	0.40∗	1.05 (0.69–1.58)	0.82∗	460
VAS-NP	1.17 (0.79–1.75)	0.42∗	0.93 (0.63–1.39)	0.74∗	454
NDI categorized	0.77 (0.50–1.18)	0.23^+^	0.80 (0.49–1.31)	0.38^+^	440
Satisfaction	0.92 (0.57–1.47)	0.72^†^	0.89 (0.55–1.46)	0.65^†^	444

PROs: Patient-reported outcomes; OR: Odds ratio; CI: Confidence interval; VAS-AP: Visual Analogue Scale for arm pain; VAS-NP: Visual Analogue Scale for neck pain; NDI: Neck Disability Index categorized. ∗continuous ordinal regression, ^+^logistic ordinal regression, ^†^binary logistic regression. Statistical significance is designated by P value < 0.05.

## 4 Discussion

We found no significant difference between degenerative cervical surgery performed by supervised residents and specialist neurosurgeons. Our data suggest that under appropriate supervision, residents can achieve outcomes comparable to those of specialist neurosurgeons. Given this study's cohort size, these findings likely reflect a true absence of clinically meaningful differences.

### 4.1 PROs and perioperative outcomes from resident-performed cervical spine surgery

Only a few studies have evaluated the impact of resident-supervised surgery on long-term postoperative PROs following cervical spine surgery (Patel et al., 2022; Galetta et al., 2020). A North American retrospective cohort study (Galetta et al., 2020) compared anterior cervical discectomy and fusion outcomes in cases assisted by either an orthopedic spine fellow or a resident, and found no differences in postoperative health-related quality-of-life scores, pain levels, or disability indices between the two groups. This study primarily shows that the effect of trainee involvement is procedural, with an increase in operative duration and intraoperative blood loss. More studies address perioperative surgical outcomes following cervical spine surgery by residents with similar results (Kim et al., 2016; Lee et al., 2018; Stienen et al., 2015; Niimura et al., 2017; Akhras et al., 2020). A majority of these studies were conducted in Anglo-Saxon countries, where independent surgical responsibility often is delayed until post-residency fellowships (Jackson et al., 2022). In another North American retrospective cohort study (Kim et al., 2016), authors analyzed 3265 cases of single-level anterior cervical discectomy and fusion from a national surgical quality registry to evaluate whether resident involvement affected short-term outcomes. After propensity score matching and multivariate adjustment, they found no significant differences in 30-day complication rates, including surgical and medical events, reoperation, or mortality between surgeries with and without resident participation. While operative time was significantly longer when residents were involved, this did not translate into increased hospital stay or adverse outcomes. These findings are consistent with our results and reinforce the safety of resident involvement in cervical spine procedures when adequate supervision and structure are in place. In contrast to our study, the residents were rarely listed as the primary surgeon. This weakens the external validity of the Anglo-Saxon studies to Germanic countries, such as Denmark, where residents act as the primary surgeon earlier in training, but always under direct supervision and only within a selected range of cases. Our results support the notion that the Germanic model, emphasizing structured, supervised hands-on training during residency, does not compromise patient outcomes, even in technically demanding procedures like cervical decompression.

### 4.2 Interpreting NDI trends and patient satisfaction across surgeon experience levels

We found a tendency toward worse NDI scores in the resident group. The sections in the NDI questionnaire are not a common indication for cervical degenerative disc disease surgeries. Therefore, the value of this outcome should be interpreted with caution. Several plausible explanations for this possible effect of educational level on NDI can be considered. Technical nuances in cervical spine surgery, such as accurate cage placement and achieving fusion in ACDF, can influence outcomes. More experienced surgeons often report better fusion rates and disability scores in cervical fusion procedures (Patel et al., 2022), suggesting surgical precision improves neck function. Post-operative rehabilitation and patient education may also play a role. Although all patients received standard follow-up, specialists might better emphasize rehabilitation or ensure patients understand activity levels. Structured post-operative rehabilitation has been linked to improved outcomes and reduced disability in spine surgery (Rahman et al., 2024; Barbosa et al., 2023).

Patient satisfaction at 1-year was similar across resident and specialist groups. Satisfaction is influenced more by communication, trust, and overall hospital experience than by surgeon seniority (Lehrich et al., 2021; Rabah et al., 2021). As both groups received comparable care, these results support involving trainees without compromising patient satisfaction (Ells et al., 2025).

### 4.3 Limitations

The retrospective nature of the study design is limited by potential errors in data collection, missing data and potential under-reporting. A large portion of patients were excluded due to missing follow-up PROs, which is a risk for potential responder bias. We assumed the missing data mechanism to be missing at random. This is supported by the observation that missingness was not strongly associated with variables typically indicative of poor outcomes. Furthermore, we included all relevant baseline covariates in the imputation model. Though the missing at random assumption cannot be formally tested, the similarity of results between complete-case and imputed analyses supports its plausibility. Limitations also include a potential selection bias in which cases were operated on by residents versus specialist neurosurgeons. Specialist neurosurgeons may have taken on more complex cases. The resident subgroup was relatively small (94 resident-performed cases vs. 370 by specialist neurosurgeons), raising the risk of Type II error. Therefore, we may conclude that any differences between outcomes are minor, as it is not possible to demonstrate in a cohort of this size. We were unable to gather detailed information on the primary surgeons' level of experience. Denmark's structured residency system with early supervised responsibility by a “see one, do one, teach one” training principle, may differ from other healthcare systems, limiting external validity. We analyzed NDI in ordinal categories (levels of disability) rather than as a continuous or change score. While this approach is valid, it inevitably loses some information.

Further studies are needed to ensure that as we train the next generation of spine surgeons, we maintain the highest standards of patient care.

## 5 Conclusion

Our results support the practice of graduated surgical responsibility: with proper supervision and case selection, resident surgeons can safely participate in cervical spine surgeries without significantly affecting patient-reported outcomes. We did not find evidence that patients who underwent surgery performed by a supervised resident had worse PROs at 1-year follow-up, indicating that simultaneous resident surgical training and patient treatment did not compromise results at 1-year follow-up.

## 6 Contributorship statement

Tobias Overmark: Designing study protocol, data collection, data analysis and interpretation, drafting the article, critical revision of the article, approval of the submitted article, corresponding author.

David Kocemba MD: Designing study protocol, data analysis and interpretation, critical revision of the article, approval of the submitted article.

Tim Damgaard Nielsen MD: Designing study protocol, critical revision of the article, approval of submitted article.

Joel Borgstedt-Bendixen MD: Data interpretation, critical revision of the article, approval of submitted article.

Mikkel Mylius Rasmussen MD PhD: Designing study protocol, data analysis and interpretation, critical revision of the article, approval of submitted article.

## Declaration of competing interest

The authors declare that they have no known competing financial interests or personal relationships that could have appeared to influence the work reported in this paper.
