# Supplementary material for: Impact of resident versus specialist performed cervical spine surgery
Source: Brain Spine. 2025 Nov 3;5:105864. doi: 10.1016/j.bas.2025.105864 (PMC12639292; doi:10.1016/j.bas.2025.105864)
Supplement: Multimedia component 1 [file mmc1.docx]

| **Supplementary A.** Floor/ceiling effects of continuous PROs and missing values for included patients (*n* = 464). | | | |
| --- | --- | --- | --- |
|  |  |  |  |
| Variable | Floor: *n (%)* | Ceiling (*n*) | Total (*n*) |
| VAS-AP baseline | 20 (4.3) | 9 (1.9) | 464 |
| VAS-NP baseline | 18 (3.9) | 8 (1.7) | 464 |
| NDI baseline | 5 (1.1) | 0 (0) | 451 |
| VAS-AP 1-year follow-up | 61 (13.1) | 7 (1.5) | 464 |
| VAS-NP 1-year follow-up | 56 (12.1) | 7 (1.5) | 457 |
| NDI 1-year follow-up | 34 (7.4) | 0 (0) | 460 |
|  |  |  |  |
| Variable | Missing: *n* (*%)* |  |  |
| European Myelopathy Scale grade | 29 (6.39) |  |  |
| NDI baseline | 13 (3.09) |  |  |
| NDI 1-year follow-up | 7 (1.44) |  |  |
| Neck Pain Duration | 5 (1.03) |  |  |
| Arm Pain Duration | 4 (0.82) |  |  |
| Satisfaction | 4 (0.82) |  |  |
| VAS-NP 1-year follow-up | 3 (0.62) |  |  |
| All other variables | 0 (0.00) |  |  |

*Note:* PROs: Patient-reported outcomes; VAS-AP: Visual Analogue Scale for arm pain; VAS-NP: Visual Analogue Scale for neck pain; NDI: Neck Disability Index.

| **Supplementary B.** Sensitivity analysis with multiple imputation for missing PROs | | | |
| --- | --- | --- | --- |
| Models | Data strategy | *n* | OR (95% CI) |
| VAS-AP adjusted^*^ | Complete case | 460 | 1.05 (0.69 – 1.58) |
|  | Imputed (MI) | 1060 | 0.97 (0.77 – 1.21) |
| VAS-NP adjusted^*^ | Complete case | 454 | 0.93 (0.63 – 1.39) |
|  | Imputed (MI) | 1060 | 0.84 (0.67 – 1.05) |
| NDI adjusted^+^ | Complete case | 440 | 0.80 (0.49 – 1.31) |
|  | Imputed (MI) | 1060 | 0.74 (0.58 – 0.93) |
| Satisfaction adjusted^†^ | Complete case | 444 | 0.89 (0.55 – 1.46) |
|  | Imputed (MI) | 1060 | 1.33 (1.00 – 1.76) |

*Note:* PROs: Patient-reported outcomes; OR: Odds ratio; CI: Confidence interval; VAS-AP: Visual Analogue Scale for arm pain; VAS-NP: Visual Analogue Scale for neck pain; NDI: Neck Disability Index; MI: Multiple imputation with predictive mean matching and seed = 123. ^*^continuous ordinal regression, ^+^logistic ordinal regression, ^†^binary logistic regression.

| **Supplementary C.** Confounding priority list | |
| --- | --- |
| Possible confounders | Priority |
| Baseline continuous PROs | 1. |
| European Myelopathy Scale grade | 2. |
| Pain duration | 3. |
| Prior neck surgery | 4. |
| ASA | 5. |
| Number of levels | 6. |
| Surgery type | 7. |
| Surgery indication | 8. |
| Age | 9. |
| BMI | 10. |
| Gender | 11. |

*Note:* Priorities of confounders were determined prior to data collection. PROs: Patient-reported outcomes; ASA: American Society of Anesthesiologists classification; BMI: Body mass index.

**Supplementary D.** Exploratory data analysis


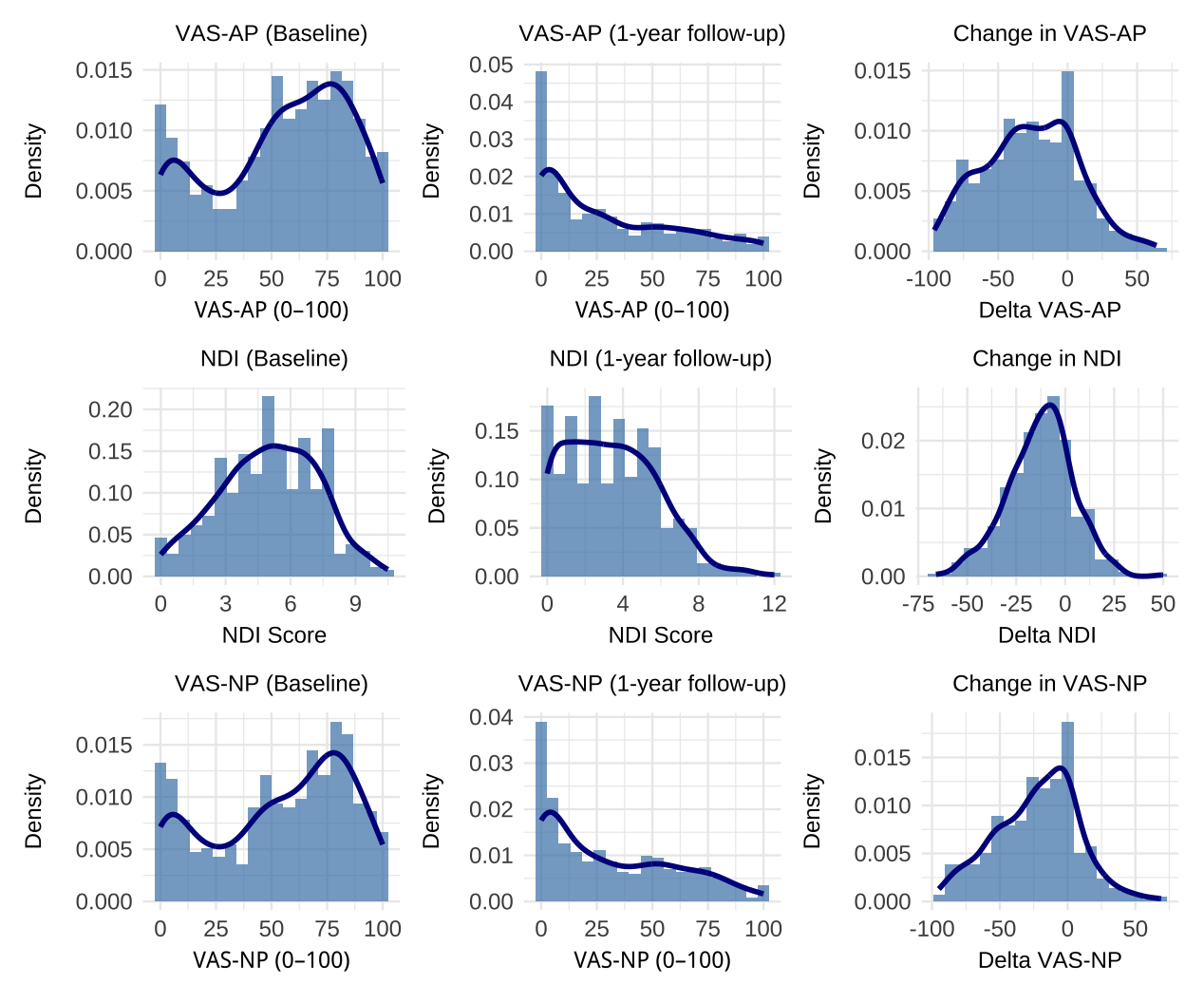


*Note:* VAS-AP: Visual Analogue Scale for arm pain; VAS-NP: Visual Analogue Scale for neck pain; NDI: Neck Disability Index.

| **Supplementary E.** Ordinal regression model diagnostics VAS-AP model | | | | |
| --- | --- | --- | --- | --- |
| Multivariate continuous ordinal regression |  | GVIF | LRT | Pr(>Chi) |
| Surgeon education level | | 1.04 | 0.007 | 0.933^*^ |
| Arm pain duration | | 1.03 | 2.922 | 0.873^*^ |
| ASA | | 1.04 | 0.014 | 0.906^*^ |
| Prior neck surgery | | 1.05 | 3.352 | 0.067^*^ |
| European Myelopathy Scale grade | | 1.08 | 2.152 | 0.142^*^ |
| Baseline VAS-AP | | 1.06 | 0.935 | 0.042^*^ |
| Number of levels | | 1.06 | 4.108 | 0.333^*^ |
| Interaction testing | |  |  |  |
| Surgeon education level × Number of levels | | |  | 0.052 |
| Surgeon education level × Prior neck surgery | | |  | 0.458 |
| Surgeon education level × ASA | | |  | 0.282 |
| Surgeon education level × European Myelopathy Scale grade | | |  | 0.885 |
| Surgeon education level × Arm pain duration | | |  | 0.478 |

*Note:* VAS-AP: Visual Analogue Scale for arm pain; GVIF: Generalized variance inflation factor; LRT: Likelihood ratio test; ASA: American Society of Anesthesiologists classification. Proportional odds assumption is assessed with LRT and ^*^Brant test.

| **Supplementary F.** Ordinal regression model diagnostics VAS-NP model | | | | |
| --- | --- | --- | --- | --- |
| Multivariate continuous ordinal regression |  | GVIF | LRT | Pr(>Chi) |
| Surgeon education level | | 1.03 | 1.357 | 0.244^*^ |
| Neck pain duration | | 1.19 | 0.822 | 0.365^*^ |
| ASA | | 1.05 | 0.017 | 0.897^*^ |
| Prior neck surgery | | 1.07 | 0.963 | 0.326^*^ |
| European Myelopathy Scale grade | | 1.08 | 6.756 | 0.009^*^ |
| Baseline VAS-NP | | 1.40 | 5.059 | 0.025^*^ |
| Number of levels | | 1.03 | >0.001 | 0.988^*^ |
| Interaction testing | |  |  |  |
| Surgeon education level × Number of levels | | |  | 0.247 |
| Surgeon education level × Prior neck surgery | | |  | 0.213 |
| Surgeon education level × ASA | | |  | 0.131 |
| Surgeon education level × European Myelopathy Scale grade | | |  | 0.477 |
| Surgeon education level × Neck pain duration | | |  | 0.290 |

*Note:* VAS-NP: Visual Analogue Scale for neck pain; GVIF: Generalized variance inflation factor; LRT: Likelihood ratio test; ASA: American Society of Anesthesiologists classification. Proportional odds assumption is assessed with LRT and ^*^Brant test.

| **Supplementary G.** Ordinal regression model diagnostics NDI model | | | |
| --- | --- | --- | --- |
| Multivariate logistic ordinal regression | GVIF | LRT | Pr(>Chi) |
| Education level | 1.01 | 0.651 | 0.722^*^ |
| European Myelopathy Scale grade | 1.04 | 3.023 | 0.221^*^ |
| Baseline NDI | 1.04 | 0.536 | 0.765^*^ |
| Interaction testing |  |  |  |
| Surgeon education level × European Myelopathy Scale grade | |  | 0.939 |
| Surgeon education level × Baseline NDI | |  | 0.105 |
| European Myelopathy Scale grade × Baseline NDI | |  | 0.417 |

*Note:* NDI: Neck Disability Index; GVIF: Generalized variance inflation factor; LRT: Likelihood ratio test; ASA: American Society of Anesthesiologists classification. Proportional odds assumption is assessed with LRT and ^*^Brant test. Events per variable ≥ 10.

| **Supplementary H.** Logistic regression model diagnostics for satisfaction model | |
| --- | --- |
| Binary logistic regression | GVIF |
| Surgeon education level | 1.03 |
| Arm pain duration | 1.67 |
| Neck pain duration | 1.73 |
| ASA | 1.05 |
| Prior neck surgery | 1.05 |
| European Myelopathy Scale grade | 1.18 |
| Baseline VAS-AP | 1.45 |
| Baseline NDI | 1.59 |
| Number of levels | 1.04 |
| Interaction testing | Pr(>Chi) |
| Surgeon education level × Number of levels | 0.223 |
| Surgeon education level × Prior neck surgery | 0.302 |
| Surgeon education level × ASA | 0.428 |
| Surgeon education level × Baseline NDI | 0.179 |
| Surgeon education level × Baseline VAS-AP | 0.673 |
| Surgeon education level × Neck pain duration | 0.512 |
| Surgeon education level × European Myelopathy Scale grade | 0.416 |
| Surgeon education level × Arm pain duration | 0.547 |
| ROC curves with area under curve (AUC) | AUC |
| Neurosurgeons | 0.644 |
| Residents | 0.640 |
| Total | 0.643 |

*Note:* VAS-AP: Visual Analogue Scale for arm pain; NDI: Neck Disability Index; GVIF: Generalized variance inflation factor; ASA: American Society of Anesthesiologists classification.

**Supplementary I.** Goodness of fit plots for continuous ordinal regression for VAS-AP (top row) and VAS-NP (bottom row) outcomes (ordinalCont package Rstudio).


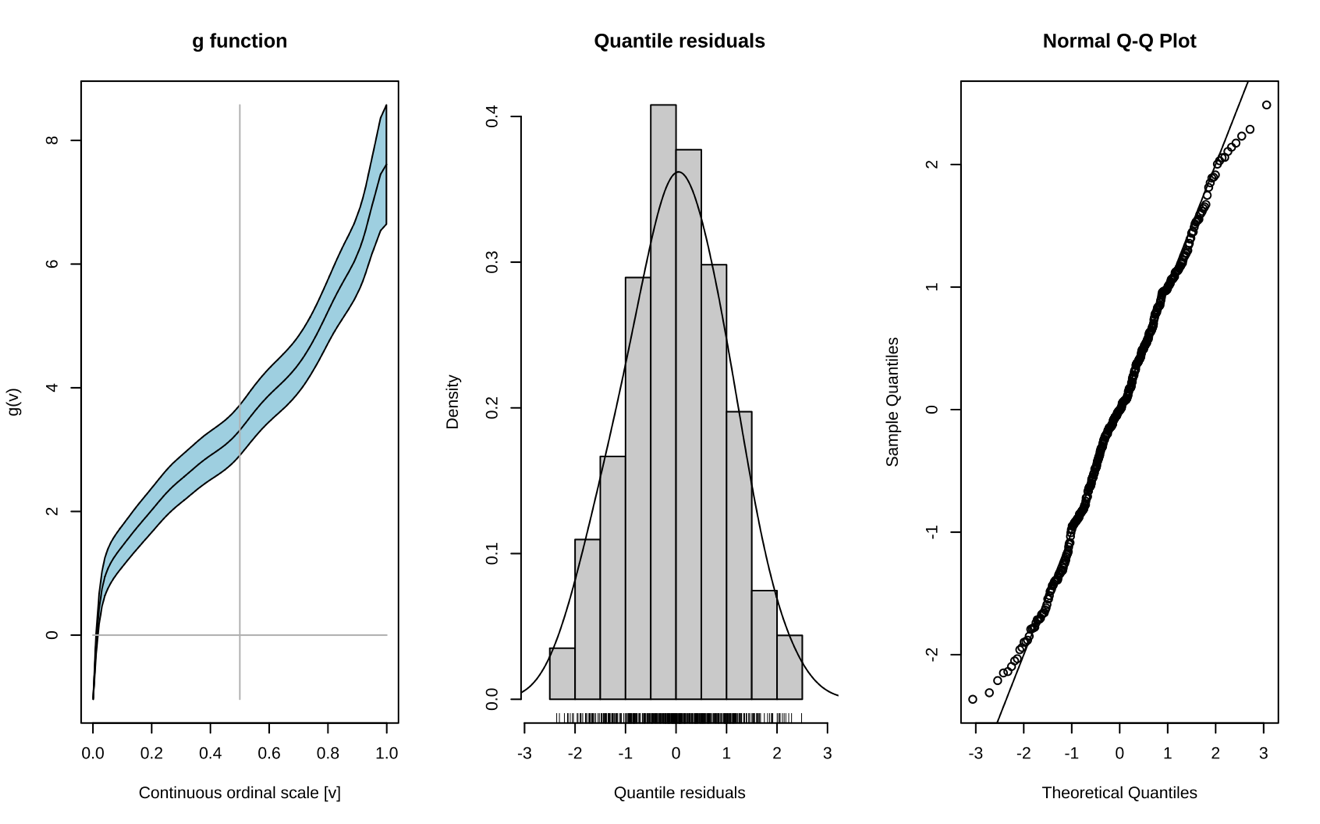

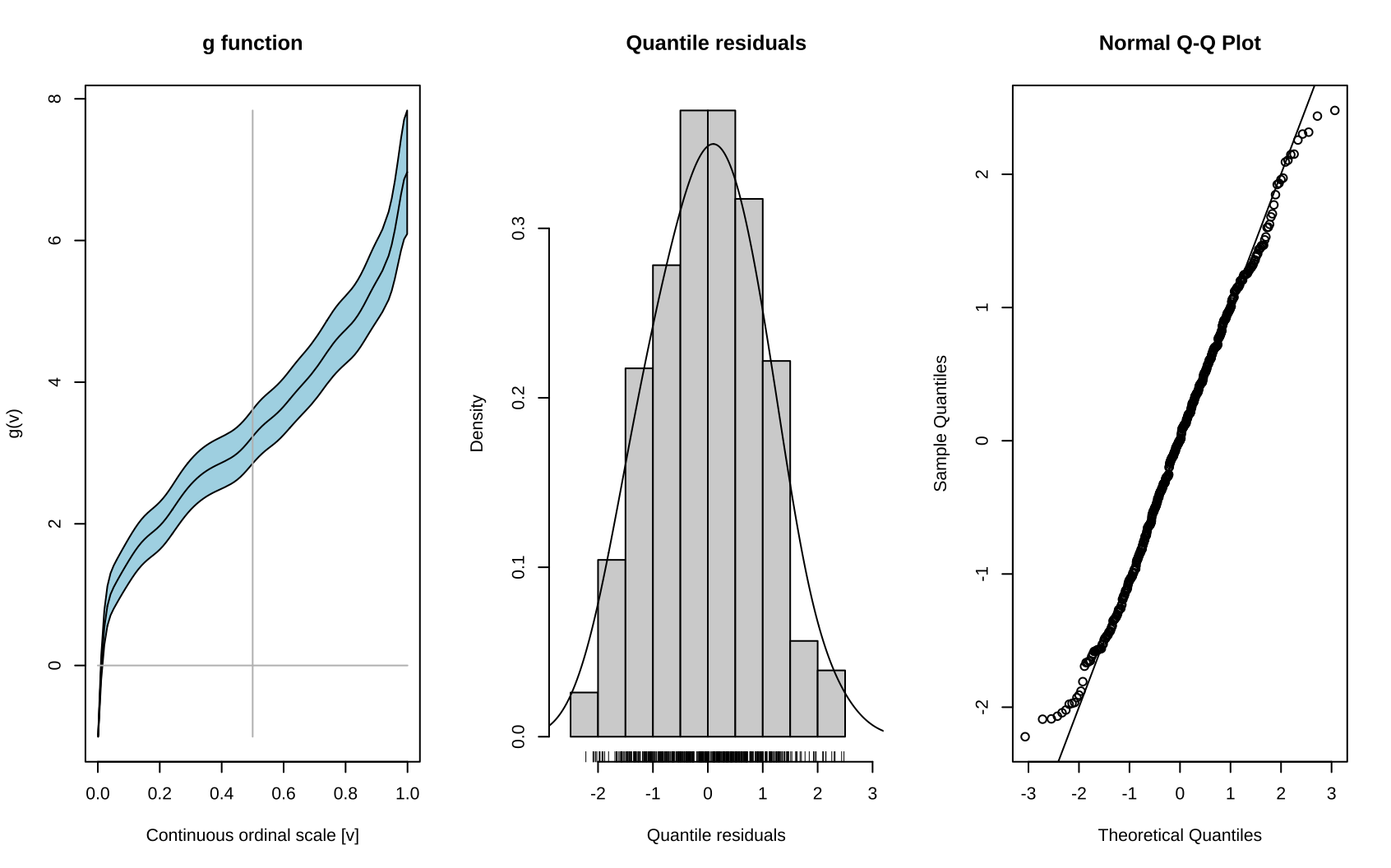


**VAS-AP model fit**

**VAS-NP model fit**

*Note:* VAS-AP: Visual Analogue Scale for arm pain; VAS-NP: Visual Analogue Scale for neck pain. Left: Estimated transformation function g(v) with 95% confidence band. Middle: Histogram of quantile residuals with overlaid density curve. Right: Normal Q-Q plot of quantile residuals. The models have 18 observations per degrees of freedom.

| **Supplementary J.** Analysis of PROs after propensity score-matching | | | |
| --- | --- | --- | --- |
| Models | OR (95% CI) | p-value | n (residents/specialist) |
| VAS-AP adjusted^*^ | 0.85 (0.51 – 1.39) | 0.51 | 94/94 |
| VAS-NP adjusted^*^ | 0.75 (0.46 – 1.23) | 0.26 | 92/92 |
| NDI adjusted^+^ | 0.65 (0.37 – 1.14) | 0.14 | 90/90 |
| Satisfaction adjusted^†^ | 0.81 (0.44 – 1.47) | 0.49 | 94/94 |

*Note:* PROs: Patient-reported outcomes; OR: Odds ratio; CI: Confidence interval; VAS-AP: Visual Analogue Scale for arm pain; VAS-NP: Visual Analogue Scale for neck pain; NDI: Neck Disability Index. ^*^continuous ordinal regression, ^+^logistic ordinal regression, ^†^binary logistic regression. Models adjusted for corresponding baseline values where applicable. 95% CIs are cluster-robust by matched subclass. Residents = treated, specialist neurosurgeons = controls.

*Supplementary J. Additional notes on matching and balance diagnostics:*

Matching algorithm: Nearest neighbor, 1:1, no replacement. Caliper: 0.20. Estimand: ATT (determined based on our research question). Overlap check: No evidence of extreme non-overlaps in any models assessed by Love plot (see below). Balance threshold: All covariates (<0.1).

*Supplementary J. Love plots*

**VAS-AP Adjusted model Love plot**


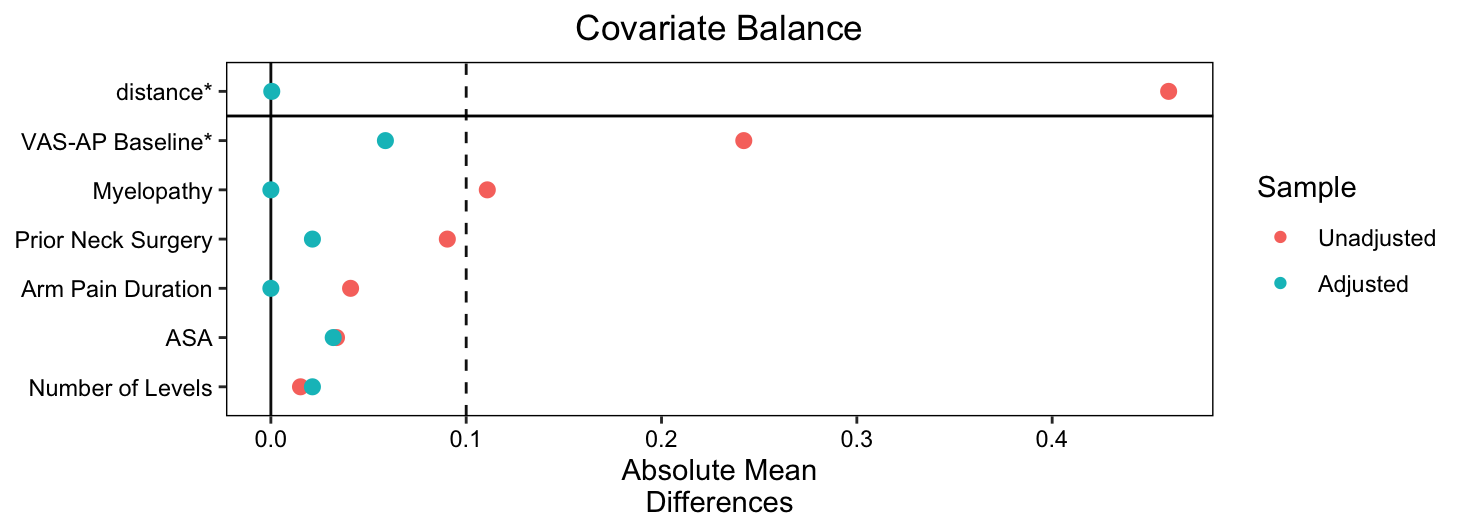


*Note:* VAS-AP: Visual Analogue Scale for arm pain; ASA: American Society of Anesthesiologists classification.

**VAS-NP Adjusted model Love plot**


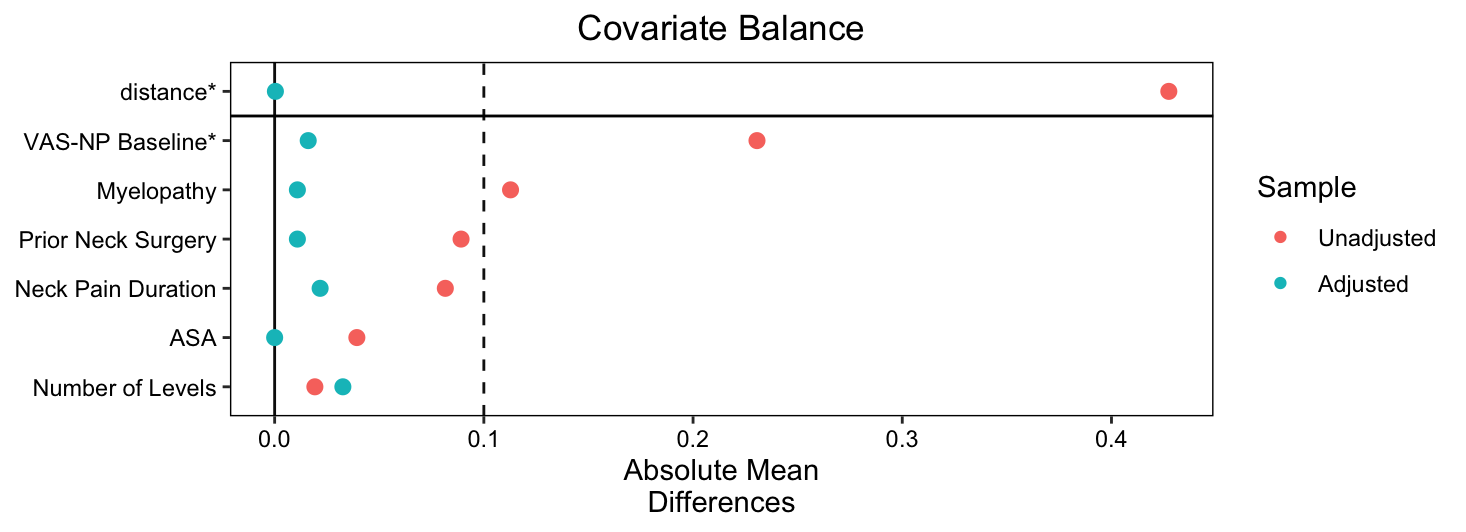


*Note:* VAS-NP: Visual Analogue Scale for neck pain; ASA: American Society of Anesthesiologists classification.

**NDI Adjusted model Love plot**


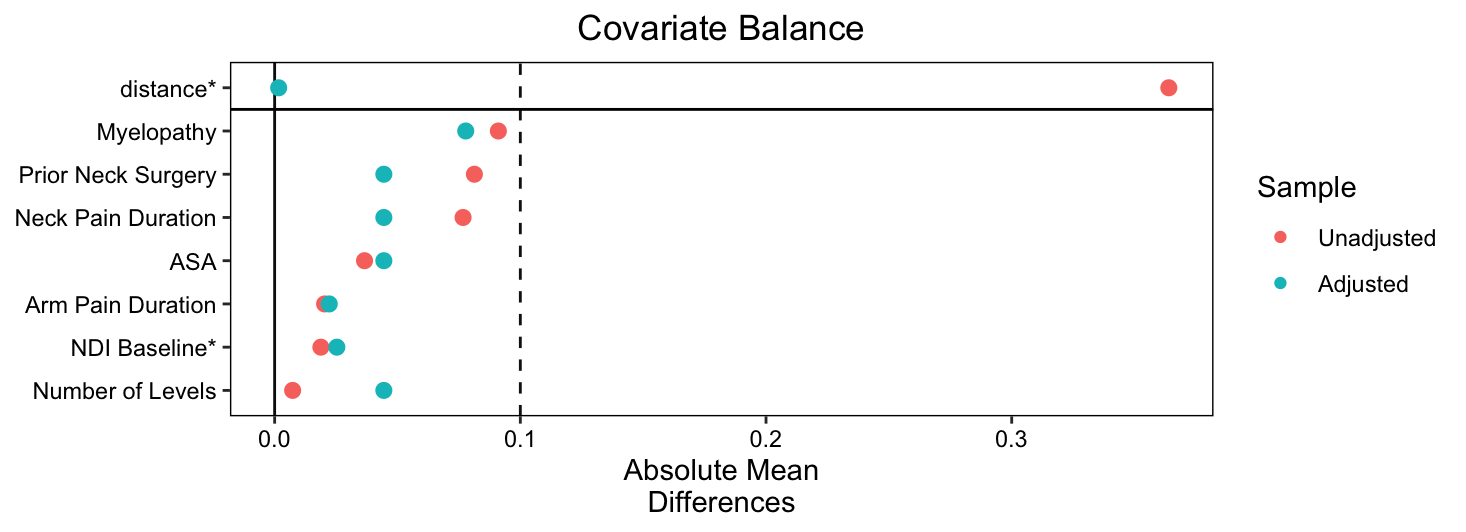


*Note:* NDI: Neck Disability Index; ASA: American Society of Anesthesiologists classification.

**Satisfaction Adjusted model Love plot**

*
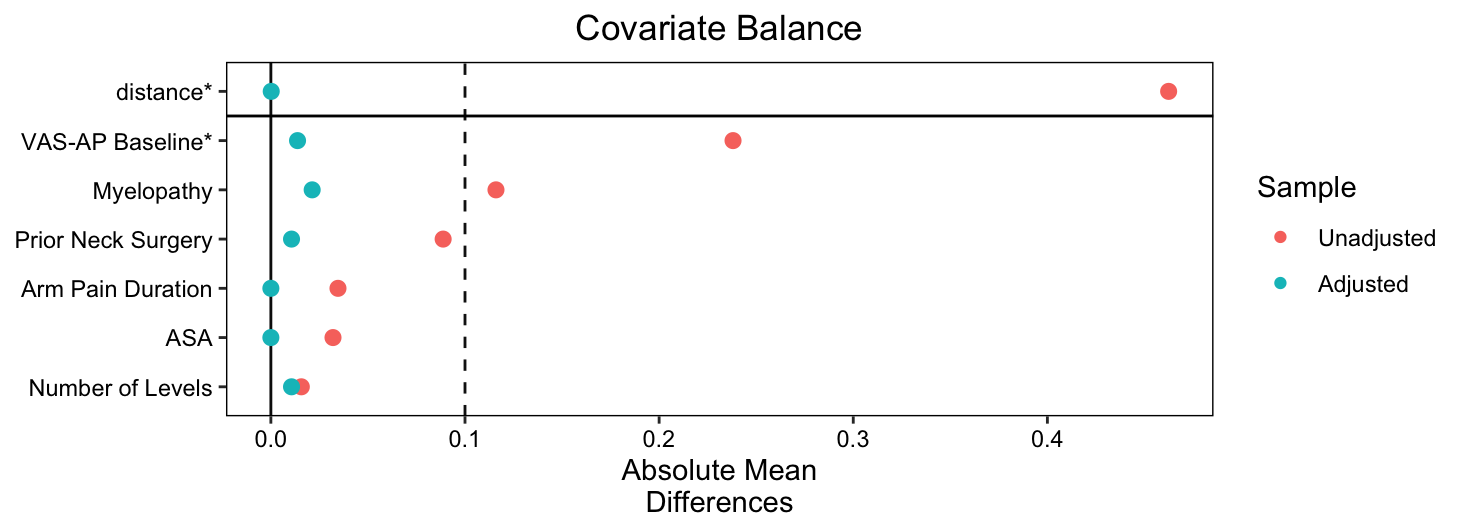
*

*Note:* VAS-AP: Visual Analogue Scale for arm pain; ASA: American Society of Anesthesiologists classification.
